# Leveraging malaria vaccines and mRNA technology to tackle the global inequity in pharmaceutical research and production towards disease elimination

**DOI:** 10.1186/s12936-024-04972-5

**Published:** 2024-05-06

**Authors:** Floriano Amimo

**Affiliations:** Faculty of Medicine, Eduardo Mondlane University, Maputo, Mozambique

**Keywords:** Drug development, Malaria, mRNA, Plasmodium falciparum, R21/Matrix-M, RTS,S/AS01, Vaccines

## Abstract

Malaria vaccine introduction in endemic countries is a game-changing milestone in the fight against the disease. This article examines the inequity in the global pharmaceutical research, development, manufacturing, and trade landscape. The role of inequity in hindering progress towards malaria elimination is explored. The analysis finds that transformational changes are required to create an equity-enabling environment. Addressing the inequity is critical to maximizing the public health impact of vaccines and attaining sustainability. Avenues to catalyze progress by leveraging malaria vaccines and messenger ribonucleic acid (mRNA) technology are discussed.

## Background

The World Health Organization (WHO) recommends childhood malaria immunization with RTS,S/AS01 (RTS,S) and R21/Matrix-M (R21) in endemic countries [1, 2]. These pre-erythrocytic virus-like particle vaccines are valuable assets in the fight against malaria with the potential to accelerate progress towards disease elimination, a longstanding global target that has, nevertheless, remained elusive for many African countries. Prior research has shown that 384.7 (uncertainty interval [UI]: 311.7–496.5) cases per 1000, 1.0 (UI: 0.7–1.6) resistant cases per 1000, and 1.1 (UI: 0.8–1.5) deaths per 1000 could be averted with the deployment of a vaccine efficacy of 40% for 10 years [3].

The introduction of vaccines in endemic countries, therefore, has the potential to revert recent unsatisfactory trends in key indicators, particularly in the context of the coronavirus disease 2019 (COVID-19) pandemic and antimicrobial resistance. Yet inequitable reliance on imported medicines by national malaria control programmes (NMCPs) in Africa may affect the supply, availability, and accessibility of the vaccines and reduce their potential public health impact on the continent.

This article examines the inequity in the global pharmaceutical landscape, from research to trade. It takes an in-depth look at the central but often neglected issues that hamper malaria elimination and eradication while delving into avenues to effectively tackle them. Drawing on current research, it first addresses inequity in essential medicines manufacturing and trade and subsequently examines hindrances to progress in research and development (R&D) in Africa. In each of these two domains, the analysis explores the factors underlying the chronic hurdles and the risks that the resulting inequity poses to the population health and sustainability of NMCPs on the continent. It moreover surveys the challenges facing the policy, strategic, regulatory, and implementation frameworks put in place to address the difficulties. Implications of the recent advances in mRNA-based therapeutics ushered in by the COVID-19 pandemic are explored.

## Manufacturing and trade

**Fig. 1:**
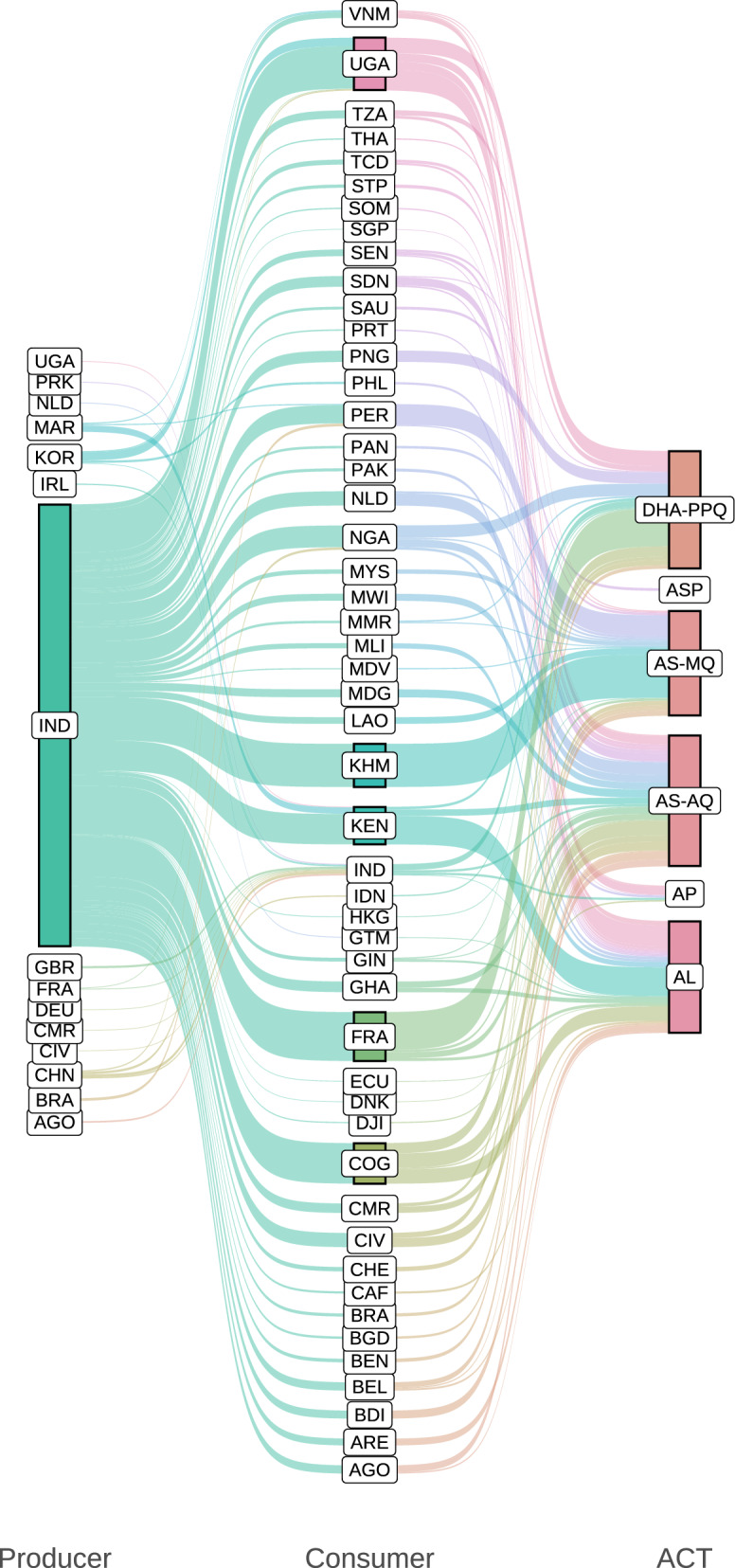
Most artemisinin-based combinations used in malaria-endemic African countries are produced outside the continent. The size of each leftmost and central node and each flow on the left and right side is proportional to the quantity of ACT medicines exported and imported by each producer and consumer country, respectively. The size of each rightmost node represents the quantity of each drug combination shipped. The colour of each left side and right side flow, as well as the leftmost and central nodes, represents each producer and consumer country, respectively. The colour of the rightmost nodes represents each drug combination. Producer and Consumer denote exporter and importer countries or territories represented by ISO 3166-1 alpha-3 codes, respectively. *ACT* medicines used for artemisinin-based combination therapy, *AL* artemether-lumefantrine, *AP* artesunate-pyronaridine, *AS-AQ* artesunate-amodiaquine, *AS-MQ* artesunate-mefloquine, *ASP* artesunate-SP, *DHA-PPQ* dihydroartemisinin-piperaquine. Data sources: [4, 5]

Reliance on imported medicines has traditionally been a major weakness of malaria control efforts in Africa. About 96–95.4% and 75.7–74.5% of global malaria deaths in 2021–2022 occurred in the WHO African Region (AFR) and among children younger than 5 years (U5) in the region, respectively [7, 12, 13]. Yet the continent has to import medicines to protect itself against the disease. Only 5% and < 1% of the medicines and vaccines Africa consumes and 3% and 0.1–0.2% of the global supply are produced on the continent, respectively [9, 10, 14]. The reliance on imported drugs also affects artemisinin derivatives used for artemisinin-based combination therapy (ACT). These are currently strongly recommended by the WHO as the cornerstone for malaria case management based on high-certainty evidence—artemether-lumefantrine, artesunate-amodiaquine, artesunate-mefloquine, dihydroartemisinin-piperaquine, artesunate-sulfadoxine-pyrimethamine (SP) (ASP)—as well as artesunate-pyronaridine [4]. Most of these ACT medicines are produced outside the continent, mostly in India [5] (see Fig. 1). This inequitable reliance on imported essential medicines perpetuates the vulnerability of national anti-malarial efforts to disruptions and shocks of global supply chains and systems, as observed at the height of the COVID-19 pandemic. This creates important risks to population health and global health security, thus acting as a structural obstacle to malaria elimination and eradication.

**Fig. 2:**
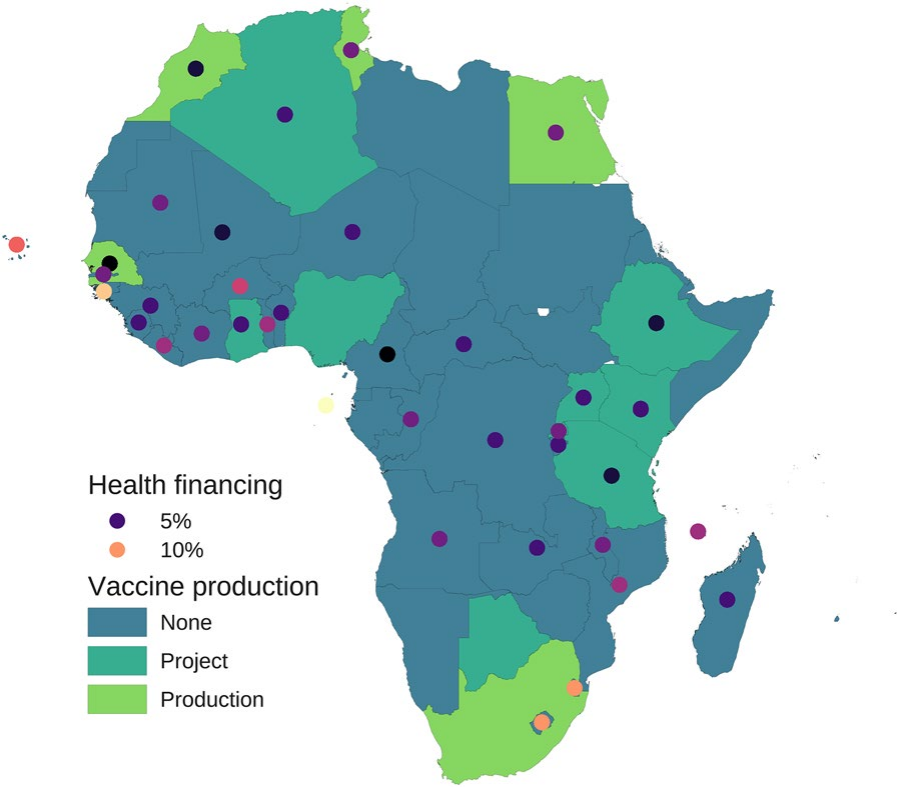
Geospatial distribution of vaccine production and health financing in Africa. Vaccine production categories shown with surface colour for each country are as follows: Production, countries with active vaccine manufacturing facilities and projects; Project, countries with vaccine manufacturing projects; None, countries without vaccine manufacturing facilities or projects. The colour of each dot is proportional to government health financing measured as the geometric mean of central government health spending as a share of general government expenditure in 2019–2021; the variation in colour intensity between or beyond the two values shown in the legend represents the corresponding variation in government health financing. Most countries do not comply with the Abuja Declaration of 2001 to allocate ≥ 15% of their annual budget to improve the health sector. Investment in vaccine manufacturing without compliance with the Abuja Declaration may result in an important diversion of government funds from the health sector, as suggested by the inverse association between the vaccine production status and government health financing observed in the current analysis (η^2^[H] = 0.17). Data sources: [6–11]

Regional and global efforts to boost local pharmaceutical production (LPP) in Africa have yielded inconsequential results. This is despite the adoption of the Pharmaceutical Manufacturing Plan for Africa (PMPA, aimed at catalyzing LPP to improve public health outcomes) in 2007 and the endorsement of its Business Plan (BP, aimed at providing approaches to accelerate the implementation of the PMPA) in 2012 [15–17]. Global inequity in drug manufacturing is also being observed with the malaria vaccines. For instance, to date, there are 18 million doses of malaria vaccines available for priority allocation in selected African countries [18]. How many of these available vaccines were manufactured in an African country? Data shows that all doses of Mosquirix, the trade name of RTS,S, used in Kenya as of 16 September 2023 were imported from Belgium [5]. This is even though some African countries have some capacity to produce vaccines nationally (Fig. 2). This status quo implies that with the expected increase in the supply of malaria vaccines as the cost decreases over time might come further reliance of African countries on imported medical products (MPs).

The COVID-19 pandemic has raised attention to the necessity to produce medicines locally or regionally and even catalyzed processes that could otherwise have taken longer to materialize. Modular mRNA production facilities have been developed by pharmaceutical companies to improve affordability and scale up accessibility of mRNA-based technologies for LPP in low- and middle-income countries (LMICs) [19, 20]. The first such a facility (‘‘BioNTainer’’, a platform for mRNA production) was set up in Kigali, Rwanda, in 2023. Just as COVID-19 ushered in the era of mRNA therapeutics and was a catalyst to install some capability for LPP in Africa, the roll-out and introduction of RTS,S and R21 on the continent could thus be leveraged to boost and scale up such LPP capability to meet the demands and accelerate attainment of universal malaria immunization coverage. However, whether, when, or how that will be attained hinges on the solidity and stability of investment in scientific, management, and financing capabilities and practices on the continent (see domain ‘‘Research and development’’). If the hindrances associated with the human component are tackled effectively and sustainably, then these facilities could become an important asset that the continent could leverage to expand its capability to produce sustainably malaria vaccines to reduce the importation and associated public health consequences.

The Framework for Action (FFA) developed by the Partnerships for African Vaccine Manufacturing (PAVM, spearheaded by the Africa Centers for Disease Control and Prevention, established by the African Union (AU) in 2021), approved by the AU in 2022, aims to enable the continent to meet 60% of its vaccine needs through local production by 2040 in the context of AU Agenda 2063 [10]. Ensuring that the PAVM-FFA does not face the same difficulties that the PMPA (adopted in 2007) and other valuable strategic and higher-level mechanisms and frameworks faced is a major challenge. Current data on the indicators established by the PMPA-BP [16]—e.g., (i) proportion of pharmaceutical market supplied by African-based manufacturers, (ii) proportion of substandard MPs in the market, (iii) number of companies achieving WHO prequalification, among others—show negligible progress. The reliance on imported medicines continues to date (see data above and Fig. 1). The percentage of substandard and falsified (SF) medicines was estimated at 5–40% and 19–50% in several countries on the continent and in the Sahel countries in 2018, respectively [21]. Furthermore, it was only in 2022 that the first African-based manufacturer, Universal Corporation Limited (Kenya), received WHO prequalification to produce SP [22]. This is an essential medicine used in the chemoprevention of malaria in pregnancy (intermittent preventive treatment in pregnancy [IPTp]) and childhood (seasonal malaria chemoprevention [SMC] and perennial malaria chemoprevention [PMC]) and as a partner drug for ACT with ASP (also see Fig. 1). It can be seen that the PMPA, despite its noble aspirations, has delivered little impact to date.

Major hurdles to these and related mechanisms and frameworks typically reside in their implementation. The rampant epidemics of corruption and mismanagement in most of the continent [23, 24] weaken not only public financing of critical infrastructure and services, but also regulatory frameworks, labour productivity, enforcement of rules, and other prerequisites for competitive LPP and trade [25–29]. The placement of unqualified or less qualified professionals in critical positions [30], a manifestation of these epidemics, lessens the impact of capacity building. Government non-compliance, e.g., with the Abuja Declaration of 2001 [6] (see Fig. 2), also a consequence of mismanagement [24], compounds the difficulties. These epidemics hinder, e.g., cross-border trade of active pharmaceutical ingredients (APIs) and sustainability of pharmaceutical investments. Thus, corruption and mismanagement are the key barriers to LPP and trade, although the nexus might not always be obvious without a rigorous analysis. Progress tracking is another challenge. An important improvement in PAVM-FFA compared to PMPA-BP is that the former has short-, medium-, and long-term key performance indicator targets [10], whereas the latter has monitoring and evaluation (M&E) indicators without targets [16]. However, for both PAVM-FFA and PMPA-BP, no baseline survey was conducted for their indicators, and research funding (e.g., to assess medicine quality) is scanty (see domain ‘‘Research and development’’). As a result, data, e.g., on compliance with pharmacopoeia requirements, is limited on the continent [21], thereby complicating the M&E of, e.g., the percentage of SF medicines nationally over time. These difficulties are far from new but are typically neglected by efforts aiming to advance LPP and trade in Africa.

Ensuring different outcomes and impacts for PAVM-FFA requires transformation, not simply incremental changes, including in business and governance practices not only across the continent but also in global organizations. A rigorous study of the root causes of chronic non-compliance by AU member states with their regulations and commitments is needed to allow its effective tackling. It is critical to leverage regional, continental, and global initiatives and organizations, e.g., the African Continental Free Trade Area (AfCFTA), World Trade Organization (WTO), United Nations Industrial Development Organization, and WHO, to reduce duplication of efforts, minimize costs, enforce compliance, overcome supply chain barriers, and ensure sustainability. Reform of the international system is necessary to strengthen the capacity of regional, continental, and global organizations to ensure the cost–benefit and sustainability of international investments and strategies to more effectively support LPP and trade in Africa. Doing so could contribute to reducing greatly historical inequities in pharmaceutical manufacturing and trade (as well as R&D). This could allow countries that most need anti-malarial drugs to produce and purchase them locally or regionally and thus remove a major obstacle to malaria elimination and eradication (Fig. 1).

In 2022, the WTO temporarily waived the Trade-Related Aspects of Intellectual Property Rights (TRIPS) agreement on MPs for COVID-19, given the exceptional circumstances of the pandemic [31]. This measure provided a critical facility for the international transfer of knowledge and technology for LPP of MPs for the prevention, diagnosis, and treatment of COVID-19. Given the epidemiological and economic burden of malaria in the AFR [12, 13, 32, 33], a similar measure could be warranted to boost LPP and accelerate progress towards a world free of malaria. To ensure sustainability and maximize impact, any TRIPS agreement waiver on anti-malarial MPs should be coupled with adequate measures to: (i) incentivize local and international drug innovation and R&D (see domain ‘‘Research and development’’), (ii) strengthen local and continental regulatory, surveillance, and quality assurance capabilities, and (iii) boost continental trade of raw materials and APIs by leveraging the AfCFTA. Making access to TRIPS agreement waivers and similar initiatives conditional on each country’s commitment and progress on these fundamental prerequisites is critical to attaining the transformational changes needed to catalyze advancement in disease control. Failure to do so could complicate the political likelihood or feasibility of a TRIPS agreement waiver for anti-malarial MPs and similar initiatives, e.g., a pandemic treaty.

Transfer of manufacturing plants to endemic countries, an asymmetric initiative, may not on its own be sustainable. African countries need to transform into an environment that disincentivizes corruption and mismanagement. This is a necessary condition to attain a competitive LPP and trade to more effectively combat their major causes of death and suffering, e.g., malaria, towards disease elimination and eradication.

## Research and development

What is the contribution of African higher education (HE) institutions to R&D to tackling the continent’s reliance on imported medicines and technology? In most of the continent, HE is not the hub for generating research, knowledge, and innovation but a neglected and underfunded sector, with research itself largely regarded as an appendage, rather than the core, of the academic work stream. Despite the commitment of AU member states in 2007 to allocate ≥ 1% of their gross domestic product in R&D [34], the continent’s public funding for R&D at 0.42% by 2019 remains one of the poorest, if not the poorest globally, just 25% of the global average of 1.7% [35, 36]. Most, if not all, of these countries, including those approved for RTS,S priority allocation, do not comply with the Abuja Declaration of 2001 to allocate ≥ 15% of their annual budget to improve the health sector [6, 37]. Among those with a combined share of global malaria mortality in 2021–2022 > 50% [12, 13], the average government health financing in 2019–2021 was ≤ 5% in the Democratic Republic of the Congo, the Niger, as well as Tanzania, with little difference in several other countries on the continent (see Fig. 2). Rarely can R&D for health take place in such a setting.

These chronic difficulties and failures of governance cannot be tackled without institutional strengthening and eradication of the rampant epidemics of corruption and mismanagement in most of the continent [23, 24]. These epidemics are also rampant in HE, affecting, e.g., research fund availability and allocation [25, 38]. Successive HE reforms implemented in Africa have failed to solve these and other core issues hindering academic R&D despite gains in other domains [39]. Poor regulatory frameworks, chronic non-compliance, inconsistent enforcement of rules, and other deficiencies have undermined the realization of the potential of reforms to tackle the root causes of the weaknesses, thereby hampering HE performance. As a result, the global inequity in R&D has lingered. For instance, even after attaining advanced academic qualifications, most African researchers remain stuck in less prominent author list positions in peer-reviewed scientific publications (in the middle) [40].

Thus, African researchers end up having a limited role in the global research priority-setting, funding allocation, cutting-edge pre-clinical research, new trial designs, setting up of trial networks, and vaccine R&D, thereby weakening the African clinical trial ecosystem [41] and the scientific productivity and competitiveness of the continent. For instance, promising research by researchers from HE institutions in Australia, New Zealand, and Japan on mRNA malaria vaccine did not involve any African researchers or academic institutions [42]. In the last 10 years, Africa filed < 1% of global vaccine patents [10]. This creates a feedback loop, thus perpetuating inequity in pharmaceutical R&D and the reliance on imported medicines and technology. Indeed, even the mRNA clinical trials ongoing in Africa, e.g., for human immunodeficiency virus (HIV, mRNA-1644, Rwanda and South Africa), are typically not spearheaded by African academic, pharmaceutical, or research organizations, but by companies based in higher-income countries [43].

In the context of chronically limited local R&D, technology importation, that is, transfer, has emerged as an avenue to accelerate tackling inequity. mRNA technology transfer initiatives have been put in motion by development partners to advance R&D in LMICs. These initiatives include the mRNA Technology Transfer Programme established around Afrigen in South Africa in 2021 by the WHO and Medicines Patent Pool to provide technology development, training, and transfer to partners in LMICs [44]. The Bill & Melinda Gates Foundation has invested or allocated approximately ≥ US$135 million in mRNA research and vaccine manufacturing technology, including $60 million allocated to Quantoom Biosciences (based in Belgium), $5 million to the Institut Pasteur de Dakar (IPD, Senegal), and $5 million to Biovac (South Africa) [45]. These initiatives are necessary to advance mRNA technology to pave the way for its use to develop medicines for major causes of death and suffering in Africa, such as malaria. They could also contribute to accelerating the reduction of the reliance of African countries on imported medicines. However, the initiatives do not include solutions to address the underlying problems [23, 24, 38] that created the need for technology transfer, such as meager research and innovation in academic institutions in most of the continent. Also, there is an important differential in funding and asymmetry in roles between Europe-based Quantoom Biosciences and Africa-based IPD and Biovac. For instance, Univercells (based in Belgium), a parent company of Quantoom Biosciences, developed a low-cost mRNA research and manufacturing technology that IPD and Biovac are expected to acquire [45]. This implies that only a part of the funds allocated to African R&D institutes may be used for R&D by them, as the other may have to be ‘‘allocated back’’ to companies from higher-income countries. Thus, these shifts in R&D can deepen inequity rather than tackle it.

Investing in establishing and strengthening research infrastructures and capabilities in HE institutions across Africa similar to those that generated the mRNA technology in higher-income countries could be more impactful and sustainable than simply transferring a mature technology for development and production. An overview of the settings under which the science that led to the mRNA technology emerged and developed can illustrate this. Building on prior work by other researchers since the discovery of deoxyribonucleic acid (DNA) by Johann Friedrich Miescher (University of Tübingen) in 1869 [46], Watson and Crick (both, University of Cambridge) in the 1950s formulated the current structure of DNA (double helix) [47]. These researchers made such a contribution working under a solid research infrastructure not dominated by corruption and mismanagement. Such a research infrastructure also allowed expansion and deepening of the understanding of nucleic acids in the subsequent decades. This allowed Karikó and Weissman (both, University of Pennsylvania), since the 1990s, to gradually unlock the therapeutic potential of mRNA—until they finally discovered that using Pseudouridine (Ψ) instead of Uridine (U) could prevent the inflammatory response and increase protein production—thus paving the way for nucleoside-modified mRNA (modRNA) therapeutics [48]. Even so, a successful mRNA vaccine was not developed until after additional research and funding, including $25 million allocated by the Defense Advanced Research Projects Agency in 2013 to Moderna [49]. In 2020–2021, as the world was under the COVID-19 global public health emergency, leveraging the accumulated science of nucleic acids, Pfizer/BioNTech and Moderna delivered the first mRNA vaccines [50, 51].

It can be seen that the game-changing discoveries that led to the mRNA technology, from Miescher to Karikó and Weissman, took place mostly at universities, which are neglected in most of Africa. Also, it took decades for the results of academic research (nucleic acids) to deliver results with pharmaceutical or clinical applicability (modRNA COVID-19 vaccines). Indeed, even malaria vaccines have been in R&D for at least eight decades. Since at least the 1940s, researchers have been attempting to induce protective immunity to malaria parasites using, e.g., killed or inactivated sporozoites, before RTS,S and R21 (that target the *Plasmodium falciparum* circumsporozoite protein and, to a lesser extent, the hepatitis B virus surface antigen) became the first and second approved human antiparasitic vaccines in 2021 and 2023, respectively [2, 52, 53]. Thus, substantive funding needs to be allocated continuously to HE institutions for research if game-changing solutions for public health challenges are to be observed on the continent. Sustainability is paramount. Otherwise, if AFR continues to neglect its HE, then even to fight against malaria (a preventable and curable disease whose approximately 3/4 of attributable deaths globally occur in its U5 [7]) the continent may have to continue relying on imported medicines and technology. Given the complexity of the biology of *P. falciparum* [54, 55], even the mRNA technology transfer, on its own, not coupled with a solid investment in HE on the continent and institutional strengthening, may not be the panacea for meager R&D, at least not as expected.

Creating an R&D infrastructure capable of replicating or surpassing the successes that resulted in the mRNA technology cannot happen under the current academic and research governance and financing systems in Africa. Thus, if the current and future technology transfer or similar initiatives are to tackle the chronic reliance by African countries on imported medicines and technology, they need to invest equally or more in transformational change to address the root causes of the chronic hindrances to progress, not only the consequences. Eradication of the neglected epidemics of corruption and mismanagement on the continent is the most sustainable pathway to accelerate the attainment of equity in pharmaceutical R&D towards malaria elimination and eradication.

## Conclusions

Transformation is needed in governance practices throughout the continent and in global organizations, as well as in the pharmaceutical landscape, from research to trade. Tackling inequitable reliance on imported medicines and technology requires solid and stable investment to establish and strengthen research infrastructures and capabilities in academic institutions on the continent. These are a necessary condition to create a sustainable environment capable of enabling endemic countries to boost innovation and LPP. Removing these major structural obstacles in the fight against malaria is critical to ensuring progress in eliminating and eradicating the disease. If these hindrances are tackled effectively and sustainably, the momentum created by malaria vaccine introduction and technological advancements ushered in by the COVID-19 pandemic could be a catalyst for bettering local research, development, and production of medicines. Lessons learned on malaria could then be translated to other vaccine-preventable diseases that, despite having effective vaccines, continue to burden the continent.

## Data Availability

The datasets used in the current study are publicly available and can be efficiently extracted from the sources cited in the article. The processed data that were presented in the main text and/or used to draw the graphs are available from the corresponding author upon reasonable request. The data analysis was performed using R version 4.3.0. Data and code sharing will require a Materials Transfer Agreement (MTA).

## Abbreviations

ACT: Artemisinin-based combination therapy
AfCFTA: African Continental Free Trade Area
AFR: WHO African Region
API: Active pharmaceutical ingredient
ASP: Artesunate-SP
AU: African Union
BP: Business plan
COVID-19: Coronavirus disease 2019
DNA: Deoxyribonucleic acid
FFA: Framework for action
HE: Higher education
IPD: Institut Pasteur de Dakar
LMIC: Low- and middle-income country
LPP: Local pharmaceutical production
M&E: Monitoring and evaluation
modRNA: Nucleoside-modified mRNA
MP: Medical product
mRNA: Messenger ribonucleic acid
NMCP: National Malaria Control Programme
PAVM: Partnerships for African Vaccine Manufacturing
PMPA: Pharmaceutical Manufacturing Plan for Africa
R21: R21/Matrix-M
R&D: Research and development
RTS,S: RTS,S/AS01
SF: Substandard and falsified
SP: Sulfadoxine-pyrimethamine
TRIPS: Trade-Related Aspects of Intellectual Property Rights
U5: Children younger than 5 years
UI: Uncertainty interval
WHO: World Health Organization
WTO: World Trade Organization
